# Divergence of X-linked *trans* regulatory proteins and the misexpression of gene targets in sterile *Drosophila pseudoobscura* hybrids

**DOI:** 10.1186/s12864-021-08267-w

**Published:** 2022-01-06

**Authors:** Alwyn C. Go, Alberto Civetta

**Affiliations:** Department of Biology, University of Winnipeg, 515 Portage Ave, Winnipeg, MB R3B 2E9 Canada

**Keywords:** Hybrid male sterility, *Drosophila pseudoobscura*, Divergent X-linked *trans*-regulatory proteins, Testes transcriptomes, *cis*-regulatory divergence, Speciation

## Abstract

**Background:**

The genetic basis of hybrid incompatibilities is characterized by pervasive cases of gene interactions. Sex chromosomes play a major role in speciation and X-linked hybrid male sterility (HMS) genes have been identified. Interestingly, some of these genes code for proteins with DNA binding domains, suggesting a capability to act as *trans*-regulatory elements and disturb the expression of a large number of gene targets. To understand how interactions between *trans*- and *cis*-regulatory elements contribute to speciation, we aimed to map putative X-linked *trans*-regulatory elements and to identify gene targets with disrupted gene expression in sterile hybrids between the subspecies *Drosophila pseudoobscura pseudoobscura* and *D. p. bogotana*.

**Results:**

We find six putative *trans*-regulatory proteins within previously mapped X chromosome HMS loci with sequence changes that differentiate the two subspecies. Among them, the previously characterized HMS gene *Overdrive* (*Ovd*) had the largest number of amino acid changes between subspecies, with some substitutions localized within the protein’s DNA binding domain. Using an introgression approach, we detected transcriptional responses associated with a sterility/fertility *Ovd* allele swap. We found a network of 52 targets of Ovd and identified *cis*-regulatory effects among target genes with disrupted expression in sterile hybrids. However, a combined analysis of polymorphism and divergence in non-coding sequences immediately upstream of target genes found no evidence of changes in candidate regulatory proximal *cis*-elements. Finally, peptidases were over-represented among target genes.

**Conclusions:**

We provide evidence of divergence between subspecies within the DNA binding domain of the HMS protein Ovd and identify *trans* effects on the expression of 52 gene targets. Our results identify a network of *trans*-*cis* interactions with possible effects on HMS. This network provides molecular evidence of gene × gene incompatibilities as contributors to hybrid dysfunction.

**Supplementary Information:**

The online version contains supplementary material available at 10.1186/s12864-021-08267-w.

## Background

Hybrid dysfunction is a prevalent form of postzygotic isolation between species [1]. Several studies have identified loci and/or genes that contribute to a reduction of interspecies hybrid fitness [2–5]. Despite the identification of single locus/gene effects, interactions are prevalent during the onset of reproductive isolation barriers [6–12]. A well characterized system of interactions involves hybrid male lethality in crosses between *D. melanogaster* and *D. simulans*. The lethality phenotype is rescuable by the *D. simulans* Lethal hybrid rescue (*Lhr*) and the *D. melanogaster* Hybrid male rescue (*Hmr*) loss of function alleles [13–15]. To rescue male viability, the interaction of these two alleles requires the absence of the *D. simulans gfzf* allele [16]. Interestingly, within species, GFZF exerts its effect as a transcriptional co-activator [17], and in hybrids between species HMR mislocalizes to sites normally occupied by GFZF with this mislocalization being rescuable by the reduced expression of the *gfzf* allele [18]. This example highlights the importance of gene × gene interactions on the onset of hybrid dysfunction and speciation. Among more closely related species, sterility is more prevalent than inviability. Crosses between *D. simulans* and *D. mauritiana* render viable and fertile females, but sterile hybrid males. Genetic mapping identified an X-linked gene known as *Odysseus* (*OdsH*) that contributes to hybrid-male sterility, but the importance of interactions is evident in that *OdsH* requires other genes to confer full sterility [2, 6]. Genome-wide surveys have supported the role of complex systems of epistasis on the onset of hybrid incompatibility phenotypes [19–22].

Sex chromosomes play a major role in speciation, as illustrated by Haldane’s rule which states that if one sex is inviable or sterile among interspecific hybrids, it is the heterogametic sex (XY or WZ) [23]. The importance of sex chromosomes on hybrid sterility and the faster sequence and gene expression divergence of X-linked genes has been established across taxa [24–31]. The large effect of sex-chromosomes in hybrid dysfunctions such as sterility could be a consequence of a drastic misregulation of sex-linked genes, sequence changes between species at *trans*-regulatory X-linked genes triggering a disruption of gene interactions that results in phenotypic dysfunction, or a combination of both. In hybrids between *D. yakuba* and *D. santomea*, X-linked recessive alleles on the X chromosome appear as significant contributors to hybrid misexpression. The expression of X-linked male-biased genes showed faster divergence and lower polymorphism between the species than autosomal genes, but hybrid male misexpression was mostly in autosomal genes [32].

*D. p. pseudoobscura* and *D. p. bogotana* is a pair of closely related subspecies that diverged from each other approximately 0.25 Myr ago [33]. In this pair, only male hybrids with a *D. p. bogotana* X chromosome are sterile. Their recent divergence makes this subspecies good candidates to study changes in *cis-trans* interaction systems that associate with early stages of reproductive isolation (*i.e*., sterility) and speciation. Mapping studies identified a major role of the X chromosome in hybrid male sterility [4, 12], but RNA sequencing revealed no evidence of any significant overrepresentation of misregulated X-linked genes in sterile relative to fertile male hybrids [34]. However, a potentially disproportional effect of X-linked *trans*-regulatory gene divergence in driving the misregulation of target genes in hybrids was suggested by the preponderance of autosomal genes with reversals in allelic expression between hybrids that matched the X-chromosome genotype [34]. Misexpression of male reproductive genes in sterile hybrids might have been facilitated by interspecies divergence in sex-linked *trans*-regulatory factors [35]. Using an introgression approach, quantitative trait loci (QTL) mapping identified one major-effect locus contributing to hybrid male sterility in the *X22-y* interval of the *D. p. bogotana* X chromosome and three other loci with small effects, in addition to a previously found major locus on the right arm of the X chromosome [4, 12]. Within the right arm locus, a gene (*Ovd*) tightly linked to the phenotypic marker *sepia* was singled out, with fertility of the hybrid rescued by a transgenic copy of the *D. p. pseudoobscura* allele [4].

Here we identify putative X-linked transcription factors within the two major sterility loci and used polymorphism and sequence divergence data from *D. p. pseudoobscura* and *D. p. bogotana* to single out amino acid substitutions that are likely to drive divergence in the expression of target genes in hybrids. The *Ovd* protein had the largest number of fixed amino acid changes between subspecies and we took advantage of its linkage to the visible *sepia* phenotypic marker to introgress, through a series of backcrosses, the fertile *D. p. pseudoobscura* allele in a sterile hybrid background. Using this genetic approach combined with transcriptomics, we identify 52 putative targets of the *Ovd* allele, of which thirty directly associate with the F_1_ hybrid sterility phenotype. The putative targets of Ovd were not located together in clusters within the genome, were enriched for peptidases, and lacked sequence divergence within proximal *cis* non-coding sequences. However, *cis*-regulatory divergence effects (*i.e., cis* only, *cis* and *trans*, and compensatory types of regulatory divergence) were detectable through the use of allele specific expression (ASE) data.

## Results

### Six proteins within X-linked HMS loci are candidate *trans*-regulatory factors with fixed amino acid changes between subspecies

We found ten protein coding genes in the right arm of the X chromosome (XR) and 203 within the left arm (XL) HMS loci. Among them, 22 had domains that could regulate gene expression (*i.e*., DNA/RNA binding) (Table 1). An analysis of sequence divergence and polymorphism identified two genes in the XR and three in the XL HMS loci with fixed amino acid substitutions between subspecies. Of the two XR genes, one (GA19787) codes for a protein with a zinc finger RNA-binding protein and the other, *Ovd*, has a MADF DNA-binding domain. The three proteins within the XL locus were GA14860, with a Broad-Complex, Tramtrack and Bric a brac (BTB) DNA binding domain, GA15499, a protein with Zinc Finger domains capable of DNA/RNA binding, and GA14176 which codes for a protein with a Pumilio RNA-binding repeat and homology domain profile (Table 1). Fixed amino acid substitutions between the subspecies can affect the way these proteins function to bind target genes and regulate their expression. However, it is also possible for non-fixed sequence changes to define specific amino acid combinations (*i.e*., haplotypes) that differentiate the two subspecies proteins and their function. To assess this possibility, we built phylogenies based on amino acid sequence alignments and used bootstrapping to determine which of the DNA/RNA binding proteins clustered the two subspecies apart. Expectedly, we found that all proteins with fixed amino acid substitutions phylogenetically separated *D. p. pseudoobscura* and *D. p. bogotana*. We also found that the GA22224 phylogeny grouped the two subspecies apart due to two different haplotypes (Fig. S1). GA22224 codes for a protein with a MADF DNA binding domain (Table 1).

**Table 1:** Proteins within the two major HMS loci that have DNA/RNA binding domains

Gene	Location	Gene/Protein Length	Protein Domain^a^	Fix NS subs^b^
	**XR_group6**:			
*GA19787*	9,483,571..9485850	2280/600	*ZF_ C3H1*	2
*GA19828*	9,485,944..9488941	2998/721	*TRUD*	0
*GA23843*	9,488,956..9494386	5431/707	BTB, ZF	0
*GA19777 (Ovd)*	9,489,069..9490357	1289/199	MADF	7
	**XL_group1e**:			
*GA14860*	5,997,848..6001444	3597/764	BTB	1
*GA15499*	5,725,119..5727373	2255/589	ZAD, ZF	1
*GA17007*	4,372,328..4374197	1870/619	bHLH	0
*GA17644*	5,558,849..5583503	24,655/710	BTB, HTH_Psq	0
*GA17691*	4,257,334..4258014	681/229	bHLH	0
*GA17723*	4,311,421..4312215	795/264	bHLH	0
*GA20959*	4,451,610..4453965	2356/424	CHROMO	0
*GA21424*	6,550,914..6554149	3236/781	ZF; MADF	0
*GA22224*	5,345,995..5347893	1899/606	ZF; MADF	0
*GA22377*	5,791,973..5794138	2166/379	FHA	0
*GA22516*	6,482,026..6484006	1981/300	ZF	0
*GA26409*	5,874,358..5892655	18,298/2679	ZF	0
*GA26473*	5,588,794..5596413	7620/533	BTB; ZF	0
*GA29095*	4,291,592..4292707	1116/371	bHLH	0
*GA12965*	4,802,268..4803405	1138/211	*RRM*	0
*GA14176*	6,018,467..6021088	2622/1375	*PUM*	1
*GA15256*	5,866,434..5869120	2687/634	*RAP*	0
*GA18065*	4,435,228..4441982	6755/500	*RRM*	0

^a^*ZF_C3H1* Zinc Finger binds mRNAs, *TRUD* Homology to the truD synthase responsible for isomerization of uridine in RNAs, *BTB* Broad-Complex, Tramtrack and Bric a brac, *ZF* Zinc Finger C2H2 DNA/RNA binding, *MADF* myb/SANT-like domain in Adf-1, *ZAD* zinc finger-associated domain, *bHLH* basic helix-loop-helix, *HTH_Psq* Helix Turn Helix (Psq type, a family named after the *Drosophila* pipsqueak protein), *CHROMO* Chromo domain signature and profile, *FHA* Forkhead-associated, *RRM* RNA recognition motif, *PUM* Pumilio RNA-binding repeat and homology domain profiles, *RAP* RNA-binding domain abundant in Apicomplexans

^b^Fixed nonsynonymous substitutions between *D. p. pseudoobscura* and *D. p. bogotana*

Most of the proteins identified experienced very few amino acid substitutions (Table 1). We used two different bioinformatics approaches to estimate the potential effect of the amino acid substitutions on protein function. Substitutions of the *D. p. pseudoobscura* protein sequence with *D. p. bogotana* amino acids variants showed no potential deleterious effects on protein function, except for Ovd (Table 2). The Ovd protein experienced the largest number of amino acid substitutions between subspecies and was the only protein with substitutions within the DNA/RNA binding domain. Three amino acid changes within the Ovd protein were highlighted by both Polyphen-2 and Provean as potentially deleterious, two of which were within the protein DNA-binding domain (Table 2).

**Table 2:** Fixed amino acid substitutions predicted to affect protein function. Scores of deleterious effects of replacing *D. p. pseudoobscura* with the *D. p. bogotana* amino acids

Gene	Substitution	Polyphen-2	Provean
*GA19787*	S310N	0.375	−0.053
	Q544L	0.001	−0.291
*Ovd*	M4I	0.009	−1.404
	**R24K**	0.574	−1.763
	**T47S**	0.991	−1.667
	**A72S**	0.574	−0.158
	A127P	0.010	0.426
	L186V	0.991	−1.632
	K190N	0.001	2.561
*GA15499*	G231R	N/A	0.224
*GA14860*	K528R	0.007	−0.339
*GA14176*	A502V	0.012	0.193

Underlined are scores for amino acid substitutions predicted as damaging protein function. Bold are amino acid changes within the DNA/RNA binding domain

### Introgression of the fertile *Ovd* allele and effects on fertility and genome-wide expression

We identified 6 DNA/RNA binding proteins within the two major HMS loci with subspecies-specific amino acid changes. Among them, *Ovd* was the most divergent protein, and also the only protein with substitutions within the protein’s DNA-binding MADF domain. Moreover, the amino acid changes in *D. p. bogotana* were deemed detrimental (Table 2). The fact that Ovd is closely linked to the tractable phenotypic marker *sepia* [4] prompted us to use a genetic introgression approach to identify whether *Ovd,* acting as a *trans*-regulatory factor, could influence the expression of target genes, and whether those changes in expression were associated with the HMS phenotype.

**Fig. 1:**
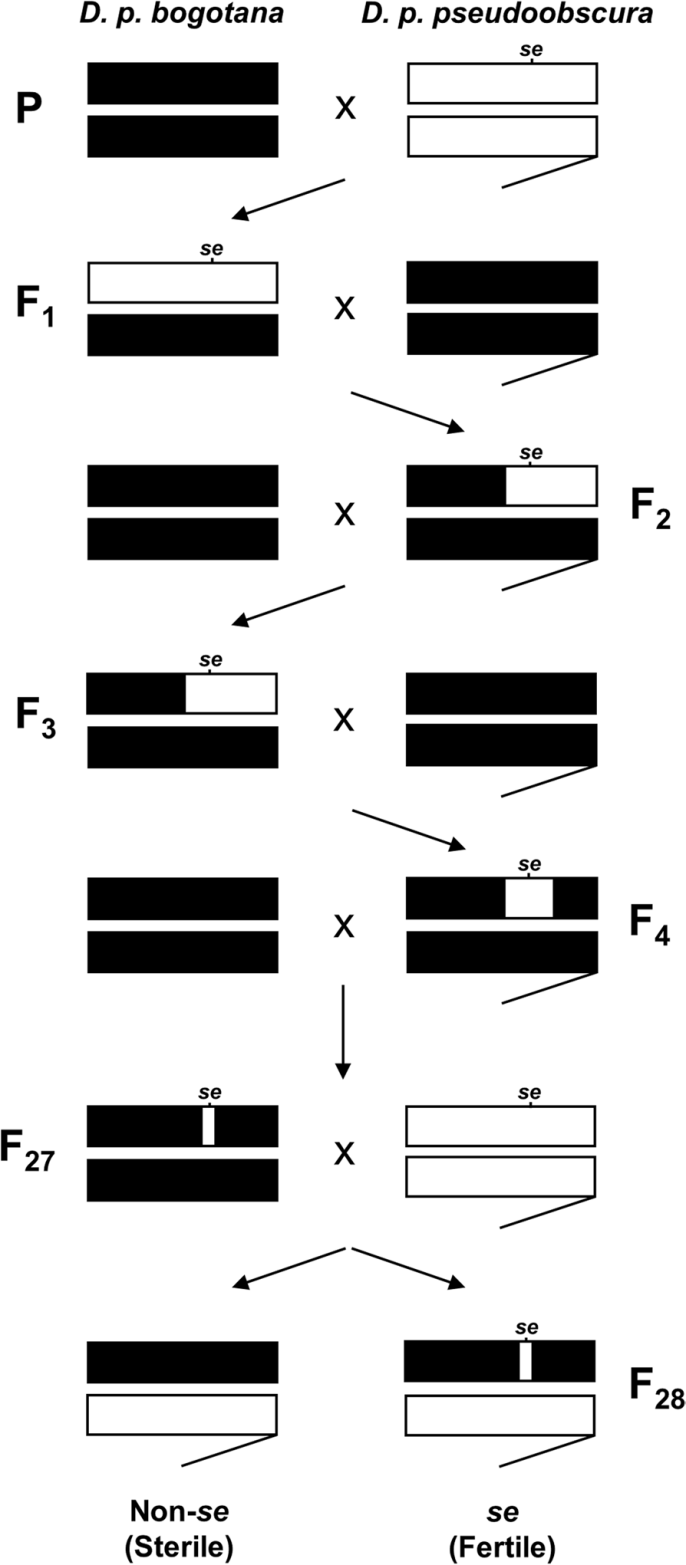
Introgression of the *D. p. pseudoobscura Ovd* allele into the *D. p. bogotana* X chromosome. The *sepia* marker (*se*), tightly linked to the *Ovd D. p. pseudoobscura Ovd* allele was introduced into the *D. p. bogotana* genetic background using an alternating mating design. *D. p. pseudoobscura* genetic content is represented by white rectangles while black segments represent *D. p. bogotana* genetic material. Only the sex chromosomes are shown in the diagram, with the Y chromosome shown as hooked bars. Recombination occurs in females which are collected in odd-numbered generations while males containing the visible marker *sepia* are selected for in even-numbered generations. Hybrids collected at each generation were backcrossed with *D. p. bogotana* to create the next generation. At the 27th generation, hybrid females were backcrossed with *D. p. pseudoobscura* males with the *sepia* mutation to create *sepia* (fertile) and non-*sepia* (sterile) hybrid males

**Fig. 2:**
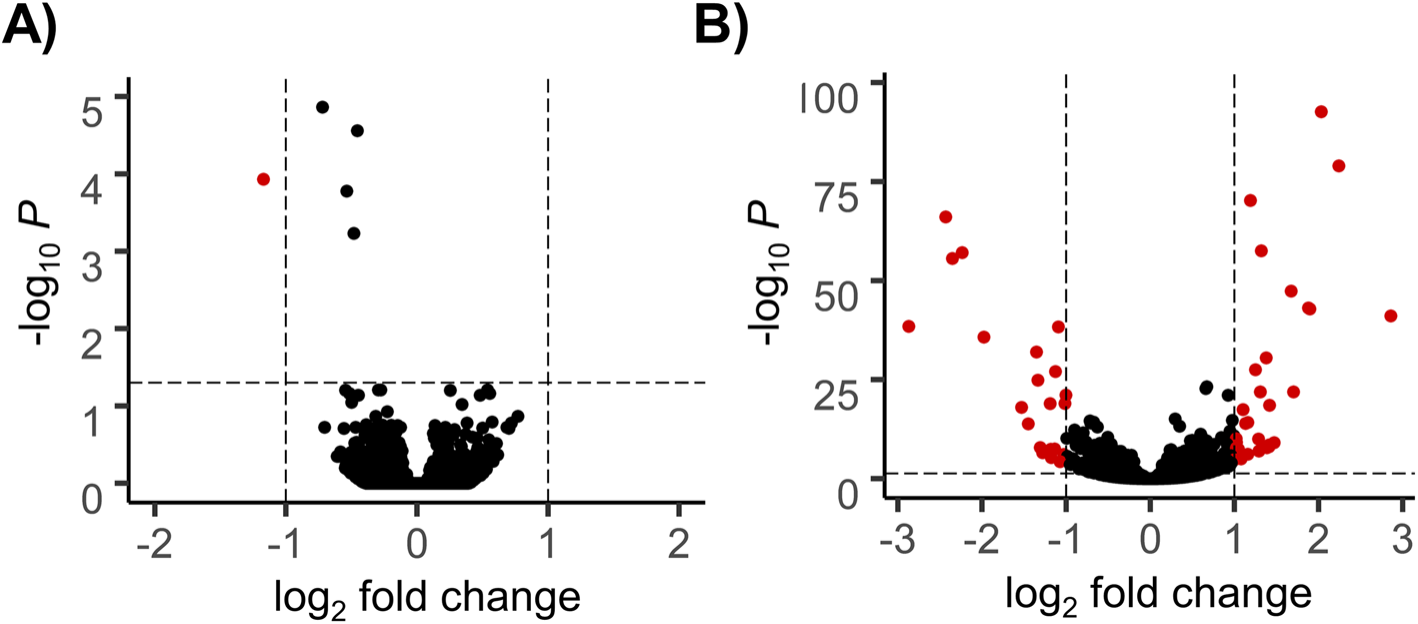
Volcano plot showing differentially expressed genes with at least a two-fold change in expression. **A** Comparison between sterile F_28_ (non-*sepia*) male hybrids and F_1_ sterile male hybrids. **B** F_28_ fertile (*sepia*) male hybrids *vs*. sterile F_28_ (non-*sepia*) male hybrids

**Fig. 3:**
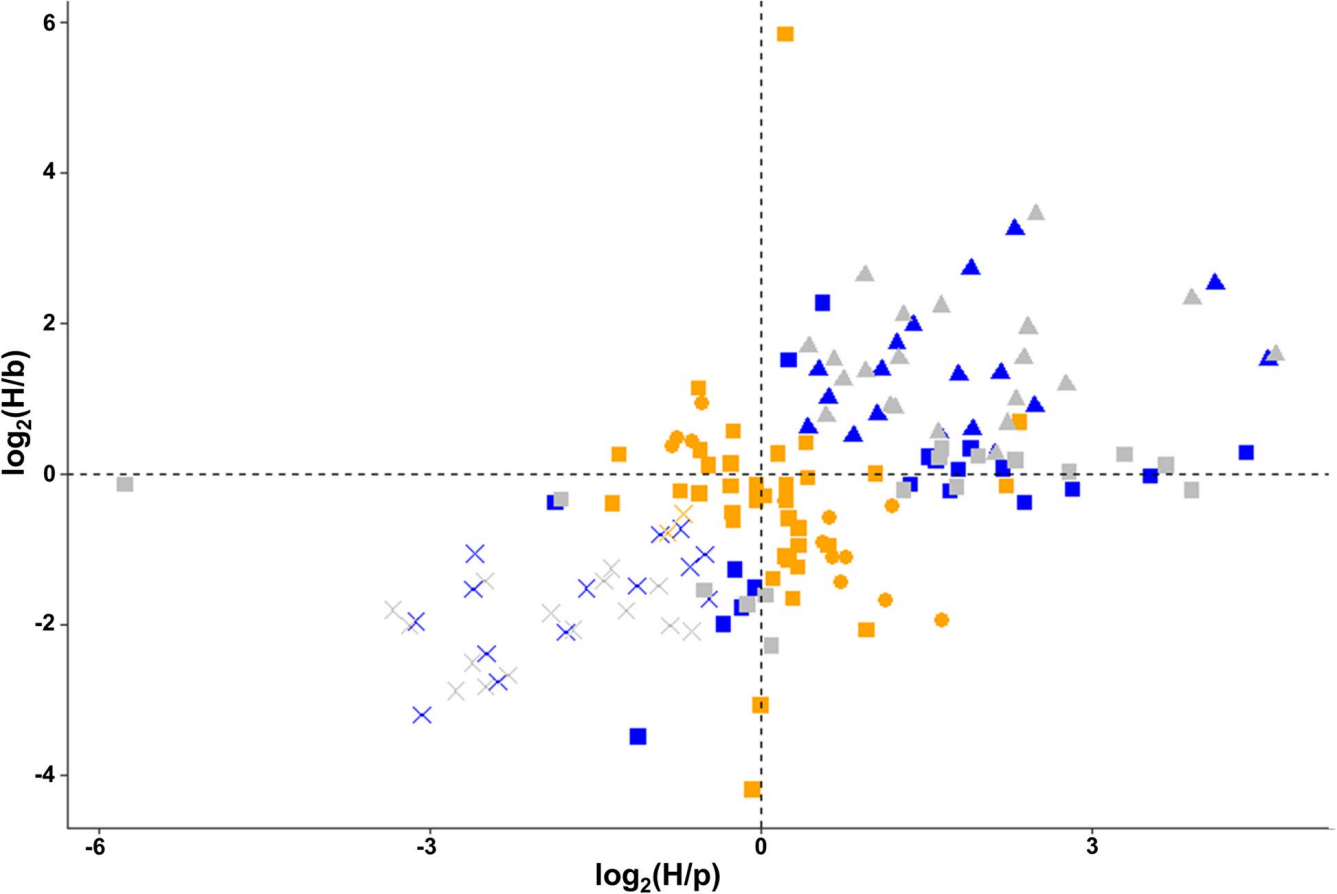
Genes differentially expressed between parental subspecies and hybrids. Gene expression in fertile F_28_, sterile F_28_ and sterile F_1_ hybrids relative to parental subspecies is shown in orange, grey and blue respectively. H = hybrids; p = *D. p. pseudoobscura*; b = *D. p. bogotana*

We took advantage of the linkage of *Ovd* with the phenotypic marker *sepia* and used a backcrossing scheme to introgress the *D. p. pseudoobscura* allele for *Ovd* into a *D. p. bogotana* X chromosome. The backcross scheme produced two types of hybrid males. F_28_ *sepia* (fertile) and F_28_ non-*sepia* (sterile) male hybrids. The non-sepia male hybrids do not contain the introgressed *D. p. pseudoobscura* allele for *Ovd* (Fig. 1) and are sterile. RNA sequencing from testes samples of the parental subspecies, F_1_ sterile hybrids, F_28_ fertile (*sepia*) and sterile (non-*sepia*) hybrids generated over 288 million reads. Approximately 205 million reads were mapped to r3.04 of the *D. p. pseudoobscura* reference genome, with an average unique mapping rate of 91.64%. All samples had nearly identical mapping rates, suggesting no mapping bias (Table S1). A comparison of the transcriptome of F_1_ sterile and F_28_ sterile (non-*sepia*) hybrid males revealed nearly identical patterns of gene expression, with only one gene showing significant differential expression between the two groups (Fig. 2A). To identify genes likely regulated by the state of the *Ovd* allele, we compared gene expression patterns between F_28_ fertile (*sepia*) and F_28_ sterile (non-*sepia*) hybrid males (Fig. 1) and found 52 genes differentially expressed between them (Fig. 2B; Table S2). When we compared the expression of these 52 genes in the F_28_ sterile relative to the parental subspecies, we found that the sterile F_28_ hybrids showed transgressive expression (*i.e.,* above or below both parental subspecies) for 36 genes with 21 overexpressed and 15 underexpressed. Only 2 genes were transgressive (underexpressed) in the fertile F_28_ hybrids relative to both parental subspecies, with 37 being non-differentially expressed and 13 additive (Fig. 3; Table S2). The number of Ovd targets with transgressive, additive, or non-differential expression in fertile and sterile male hybrids is significantly different (Table S2; 2 × 3 Fisher Exact test: *P* < 0.001), with the sterile hybrid showing significantly more transgressive genes and less additive and non-differentially expressed genes (Table S2; 2 × 2 Fisher exact test; P < 0.001).

To further classify which of the potential targets of Ovd were likely directly associated with hybrid male sterility, we identified genes with transgressive expression in the F_1_ sterile males relative to the fertile groups (*i.e.,* the fertile F_28_ male hybrids and both parental subspecies). We found that among the 32 genes that fit this category, 30 had shared transgressive expression with the F_28_ sterile male hybrids (Table S2).

### Chromosomal distribution of Ovd targets and Gene Ontologies

One of the 52 targets of Ovd is unmapped (GA32052). Among the others, 10 mapped within the second, 11 to the third, 11 on the fourth and the remaining 19 to the X chromosome. We found no significant differences in the proportion *per* million bases of targets of Ovd across chromosomes (χ^2^ = 3.80; df = 3; *P* = 0.28) or between X *vs*. autosomes (χ^2^ = 0.06; df = 1; *P* = 0.81). However, we found a non-random distribution of the 30 sterility targets across chromosomes (χ^2^ = 8.31; df = 3; *P* = 0.04) and a significantly higher proportion in autosomes than the X-chromosome (χ^2^ = 6.13; df = 1; *P* = 0.01) (Table 3A; Table S3). Autosomal HMS QTLs were previously identified in the 2nd and 3rd chromosomes [12] and we have previously mapped some male-reproductive genes to these QTLs as uniquely misregulated in the F_1_ sterile hybrid condition [34]. Here we further validate genes GA17404, GA20583 and GA20811 as putative targets of Ovd with a role in HMS (Table S3).

**Table 3:** Distribution of Ovd targets across chromosomes (A), and Ovd gene targets clustered within approximately 5Kb (B)

A)				
Chr	Tgt	Chr_length (in million bp)		Prop_Tgt
X	19 (6)	49.5		0.38 (0.12)
2	10 (6)	30.8		0.32 (0.19)
3	11 (7)	19.8		0.56 (0.35)
4	11 (10)	27.2		0.40 (0.37)
Unmapped	1			
	**Prop_Tgt**			
X	0.38 (0.12)			
Autosomes	0.41 (0.30)			
B)				
Clusters	Chrom	Position	Dist	Orthologs (prob)
Dpse\GA20266 Dpse\GA12467	XR_gr6	11,006,949..11001889 11,012,793..11008200	1251	CG7330 (7.46E^−97^) CG13699 (0)
Dpse\GA30092 Dpse\GA30093	2	2,654,310..2653746 2,658,297..2657180	2870	CG42827 (2.98E^−09^); CG42828 (2.24E^−09^) CG42827 (1.49E^−12^)
Dpse\GA32028 Dpse\GA32735	3	17,507,910..17507170 17,511,839..17511279	3369	NA NA
Dpse\GA12058 Dpse\GA12057	4_gr3	433,681..433000 441,275..438883	5202	CG13117 (1.49E^−28^) CG13116 (2.98E^−87^)

*Tgt* Number of gene targets, *Prop_Tgt* The number of gene targets per chromosome length (Chr_length). Number in parenthesis are for sterility targets.

Dist is the distance (in bp) between genes. Prob is the probability of random hit (E-value × size of nr database)

Within chromosomes, we found only four gene clusters of Ovd targets. These clusters had mostly two gene targets, with at least one of the two identified as a sterility target (Table S3). To identify *D. melanogaster* genes with sequence similarity, we performed BLASTp searches using the genes within clusters as query against the GenBank nucleotide (nr) database. Given the size of the nr database, we only kept hits with e-values lower than 2.7E^−11^ so that the probability of getting an alignment with greater similarity due to chance was lower than 0.01. The genes on the X, third, and fourth chromosome clusters returned no hits or hits to uncharacterized *D. melanogaster* genes (Table 3B; Table S4). The second chromosome cluster contained GA30092 and GA30093, with GA25574 nested in between them. GA25574 is also a significant target of Ovd when the statistical detection threshold is lowered to a log_2_ fold change (lfc) higher than 0.5. These three genes consistently returned *D. melanogaster* genes *CG42827* and *CG42828* (Table 3B; Table S4). CG42827 and CG42828 are located in chromosome 3R of *D. melanogaster*, which is syntenic to the *D. pseudoobscura* second chromosome (Muller element E [36];). Moreover, these two *D. melanogaster* genes are only 103 bp from each other and are known to be targets of the testes meiotic arrest complex (tMAC) [37, 38].

**Fig. 4:**
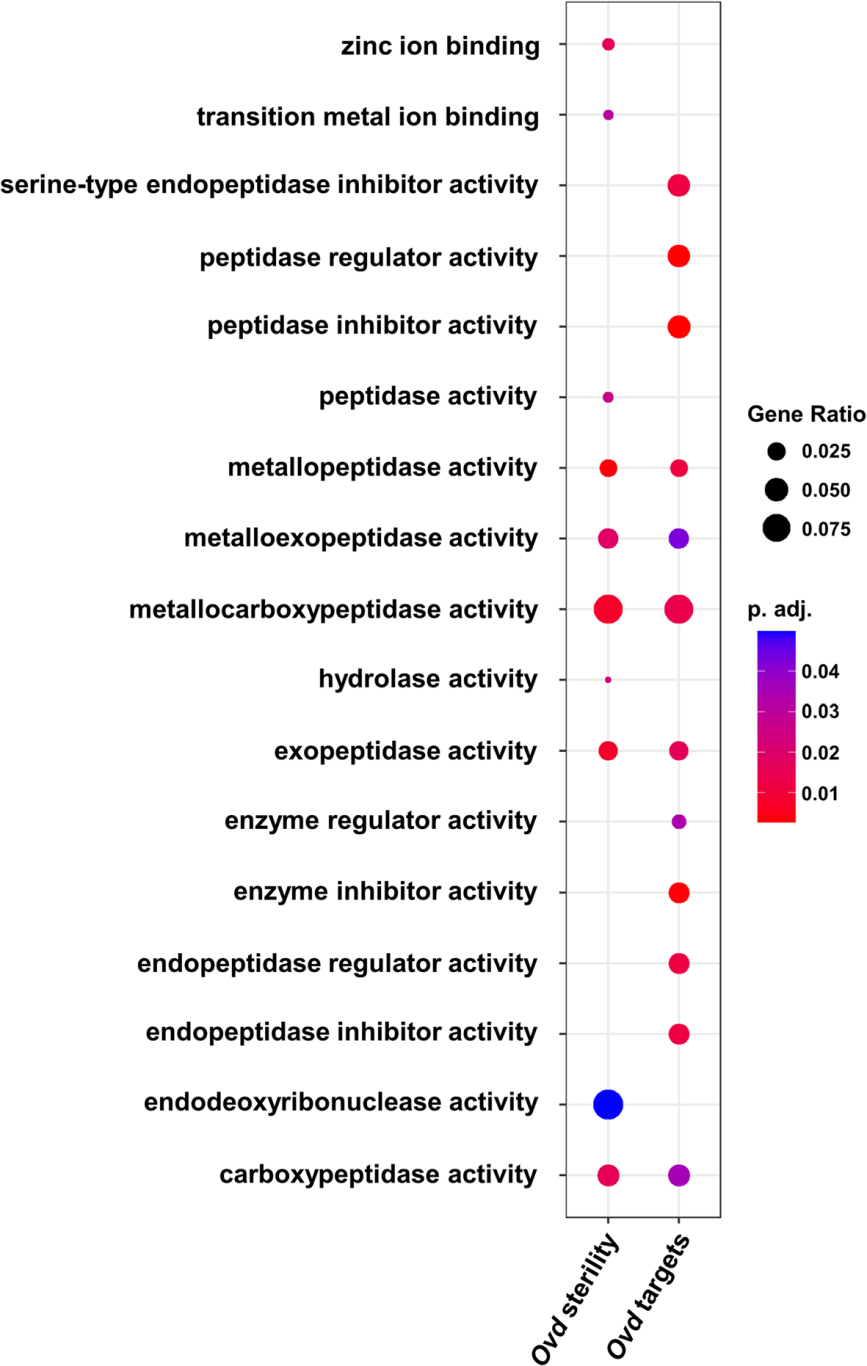
GO term enrichment analysis of targets of Ovd and targets linked to sterility. The size of the dots in the dot plot are proportional to the gene-class ratio and the dots are colored based on the FDR-corrected p-values

The genes within the second chromosome cluster were serine-endopeptidase inhibitors that we have previously highlighted as possible sterility genes in an RNA-seq analysis of the entire male reproductive tract of F_1_ hybrids and a follow up qPCR assay of expression of candidate HMS proteases in backcross males [34, 39]. Here we also searched for significant enrichment of Gene Ontology terms using g:GOST within g:Profiler [40] among Ovd targets and found enrichment for peptidases (Fig. 4).

### Targets of Ovd and *cis*-regulation

**Fig. 5:**
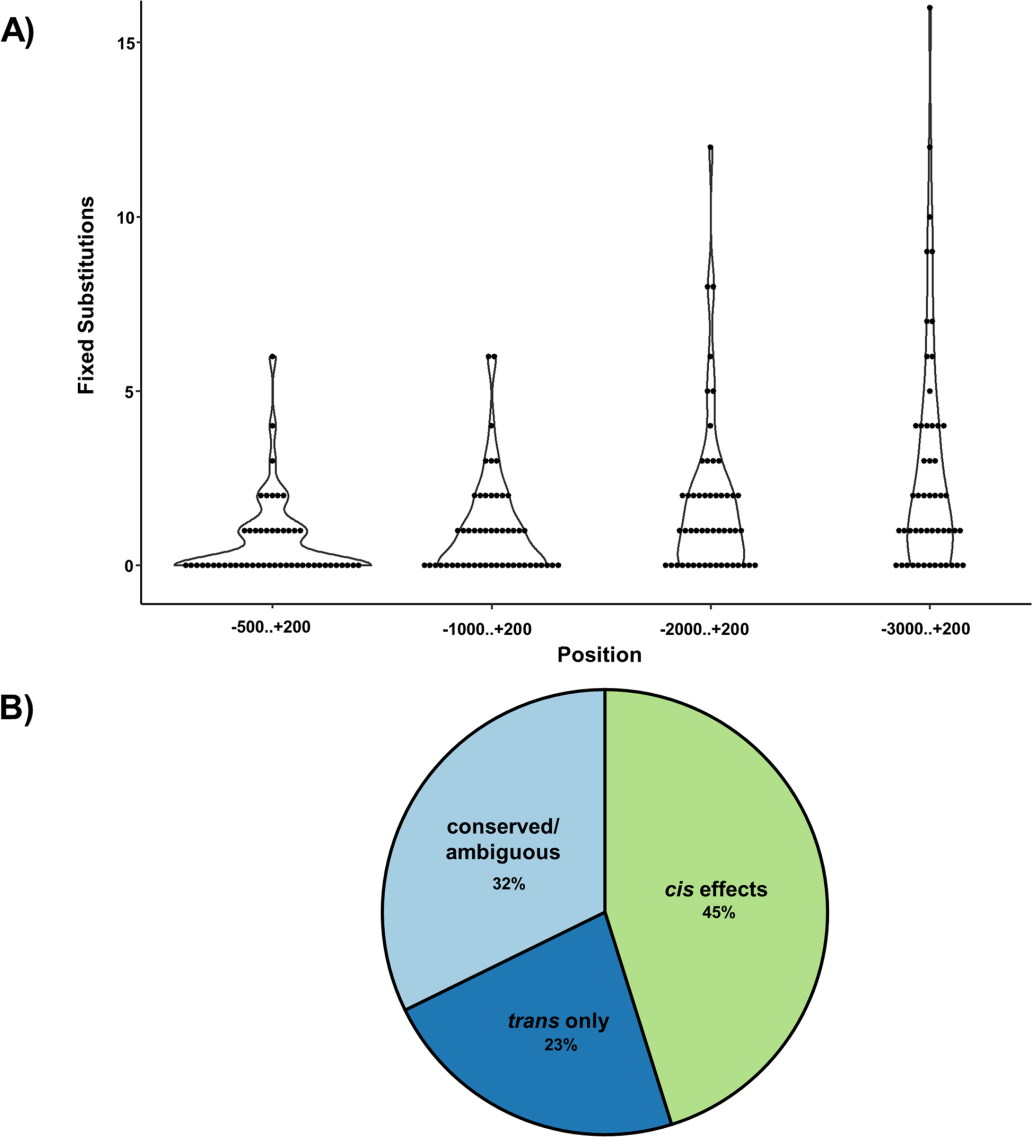
Divergence at targets of Ovd. **A** Divergence as number of fixed substitutions between subspecies at non-coding sequence positions relative to the transcription start site (TSS). **B** Percentage of conserved/ambiguous, *cis* effects, and *trans* only regulatory divergence between subspecies. “*cis* effects” is a broad category for genes with any form of *cis-*regulatory divergence. This includes *cis* only, *cis* and *trans*, and compensatory types or regulatory divergence

The Ovd protein has three out of seven fixed non-synonymous differences between *D. p. pseudoobscura* and *D. p. bogotana* located within its MADF DNA-binding domain making it a putative divergent *trans*-regulatory element between the subspecies. Of the 52 targets, sequence data was available for both subspecies except for an unmapped singleton (GA32052). For all the remaining 51 targets, we examined proximal *cis* sequence divergence between the subspecies using three approaches. Counts of the number of fixed nucleotide substitution between subspecies found a higher number of fixed changes when we considered longer sequence regions upstream of the transcription start site (TSS), but overall, there was limited evidence of proximal *cis* sequence divergence (Fig. 5A). We found, on average, 1 fixed change 1000 bp upstream and only 3 changes 3000 bp upstream of the TSS (Table S5). A couple of genes were outliers, particularly GA24794 and GA32735, with 6 and 4 changes respectively in the more proximal (−500 to +200) promoter gene region (Fig. 5A; Table S5). We also looked for nucleotide changes within putative MADF binding sites (*i.e*., Adf-1) identified using PROMO. We found that all gene targets had Adf-1 sites, with an average of about 12 sites per gene (Table S5). However, only three genes (GA31589, GA18350 and GA23864) had one fixed nucleotide change within an Adf-1 site, with gene GA18140 being the only other gene for which combinations of polymorphic sites at Adf-1 locations also distinguished the two subspecies (Table S5). Overall, like with random fixed nucleotide changes, the result shows a very small number of genes with nucleotide substitutions within putative binding sites that differentiate the *D. p. pseudoobscura* and *D p. bogotana* proximal *cis* regions. These approaches, whether based on counts of overall or localized nucleotide changes, are limited due to the uncertainty of whether such nucleotide changes can truly affect the binding of *trans*-regulatory factors and cause misregulation. Nevertheless, they do show a very limited number of genes (6 out of 51; 11%) with changes in *cis* regions proximal to the TSS.

Finally, we also employed single nucleotide polymorphisms (SNPs) from parental subspecies to identify allele specific expression (ASE) in the hybrids and infer the contributions of *cis*- and *trans*-regulatory divergence to gene misregulation. The main goal of this analysis was to compare the proportion of proximal *cis* sequence divergence estimated from sequence data to the proportion of *cis* effects detected using ASE. The patterns of allele expression between the parental subspecies and between the parental alleles within the hybrid background can be used to infer different types of *cis-* and *trans-*regulatory divergence (see Methods) since *cis*-regulatory elements affect gene expression in an allele-specific manner, while parental alleles in a hybrid background are in a common *trans*-acting environment. This approach, while widely used, is dependent on the availability of informative SNPs which are often limited in comparisons between closely related subspecies. Thus, we only had SNP information for 31 of the 51 target genes. Among the 31 targets, 45% show some form of divergent *cis* effect which includes *cis* only, *cis* and *trans*, and compensatory types of regulatory divergence (Fig. 5B; Table S5). While these estimates can be biased by the availability of informative SNPs, there is a clear difference between the paucity of proximal (*e.g.,* 3000 bp upstream) *cis* sequence divergence and the detection of *cis*-regulatory effects on expression using ASE analysis, which captures any *cis* effect regardless of proximity to the transcript being regulated.

## Discussion

Changes in gene regulation can contribute to phenotypic changes and influence evolutionary trajectories [41–43]. The role played by *cis*- and *trans*-regulatory elements in gene expression divergence and speciation has been extensively studied [35, 44–47], but it is not well-known how interactions between *cis*- and *trans*-regulatory elements might contribute to speciation. The use of closely related taxa in early stages of species differentiation, like the subspecies pair *D. p. pseudoobscura* and *D. p. bogotana*, offers an opportunity to explore the role of such interactions in gene expression divergence related to the well-established HMS postzygotic barrier between this subspecies pair. Moreover, previously mapped HMS loci and genes associated with the HMS phenotype [4, 12] allowed us to identify putative targets of an HMS *trans*-regulatory protein for the first time, using a combined approach of classical genetics, transcriptomics, and bioinformatics.

The fact that a previous analysis of misexpressed male reproductive genes revealed no over-representation of misregulated X-linked genes in hybrids between *D. p. pseudoobscura* and *D. p. bogotana* [34] and that major HMS loci were mapped on the X chromosome [12] led us to focus on X-linked divergent proteins within HMS loci. Overall, we identified several possible *trans*-regulatory X-linked proteins within previously mapped HMS loci and focused on Ovd, due to the number of fixed substitutions within its DNA-binding MADF domain and the availability of a phenotypic-linked marker for allele swapping between subspecies. We found 52 putative targets of Ovd with misregulated expression in sterile backcross males and the result offers a glimpse into a gene regulatory network with possible implications in early species divergence. We acknowledge that we cannot fully rule out the possibility of having introduced other unselected sterility loci along with Ovd, but given the number of generations used in the introgression approach we believe this is unlikely. Interestingly, the 52 targets were randomly distributed, but targets more likely linked to sterility were over-represented in the autosomes. The effect of a divergent protein between subspecies, like Ovd, on the expression of autosomal gene targets highlights not only the role of the X chromosome but is also consistent with the prevalence of interactions between sex chromosomes and autosomes in HMS [48–50]. The control of gene expression is complex and its regulation can be compartmentalized into gene clusters with shared chromatin domains and similar patterns of expression [51, 52]. While we did not find an extensive number of gene clusters among misregulated gene targets, we identified a few spread across different chromosomes. One particularly interesting cluster contained GA30092, GA30093 and GA25574. These three genes are orthologs of *D. melanogaster* genes known to be targets of the testes meiotic arrest complex (tMAC) proteins which regulate the transcription of genes required during spermatid differentiation [37, 38]. This suggests disruption of late sperm development in hybrids that, in agreement with the sterility phenotype described for hybrids between the *D. pseudoobscura* subspecies pair, does not affect sperm production but can impair sperm form and function [53–55].

In agreement with our previous results from assays of misexpression of male reproductive genes in sterile F_1_ hybrids between *D. p. pseudoobscura* and *D. p. bogotana* [34], we find an over-representation of testes-expressed peptidases among targets of Ovd. Proteases have been found to play important roles in sperm development. In mice, protease serine 50 is required for proper head-tail formation and its effect might be through the mediation of heterochromatin maintenance [56]. In lepidoptera, proteases play an important role in both the acquisition of motility of parasperm and the eusperm bundle dissociation [57, 58]. Proteases are also important for proper sperm motility in a wide variety of species ranging from nematodes [59] to humans [60, 61]. Thus, while our results might appear contradictory to those that have reported an overrepresentation of misregulated spermatogenesis genes in *Drosophila* sterile hybrids [62–64], it likely reflects on the developmental defects manifested by the different hybrids. The lack of spermatogenesis genes among targets of Ovd in sterile males is in agreement with the noticeable lack of developmental defects in the sperm of *D. p. bogotana* × *D. p. pseudoobscura* sterile hybrids [53–55]. Our finding of significant overrepresentation of proteases among targets of Ovd brings forward the hypothesis that Ovd might exert its action through an alteration of expression of proteases of yet unknown function, but likely capable of influencing aspects of sperm morphology and physiology such as head-tail formation and sperm motility.

Our results show limited evidence of sequence divergence in proximal non-coding regions despite ASE divergence that supports *cis* effects in regulation. It is therefore likely that *cis*-regulatory effects are exerted through distant regulatory binding sites, like silencers and enhancers. One possibility is that Ovd might affect gene expression through modification of heterochromatin, as suggested by models that implicate satellite DNAs in speciation [65–67]. There are reasons to entertain this as a likely explanation. First, the well-characterized HMS gene *OdsH* encodes for a transcription factor that exerts its sterilizing role by differentially binding heterochromatin and causing its decondensation [2, 68]. Satellite DNA found in heterochromatic regions can perpetuate themselves through meiotic drive while affecting male fertility [69, 70]. In fact, the *D. p. bogotana* allele of *Ovd* is not only involved in HMS but also segregation distortion of the X chromosome through meiotic drive, with both phenotypes involving the same regions of the X chromosome [4, 12]. A common genetic basis creates a possible situation of genetic conflict which can fuel the faster evolution of the speciation protein Ovd between these two closely related subspecies. Second, the over-representation of misregulated peptidases might have implications on chromatin remodeling. For example, germ cell nuclear acidic peptidases (GCNA) are proteins containing an intrinsically disorder region (IDR) which is important in the creation of nuclear structures for the assembly of protein-nucleic acid complexes that control chromatin structure and transcription [71, 72]. GCNA may exert its function through their Spartan domain. This domain resembles metalloprotease and zinc finger domains, and there is evidence that Spartan proteins cleave DNA-protein cross-links causing modifications that interfere with chromatin remodeling, DNA replication and transcription [73–75]. Recent work shows that mutations of GCNA results in genomic instability in *Drosophila melanogaster* and these peptidases are important for preventing segregation defects [72]. While the connection is speculative, it is interesting to note that the complex of Ovd targets includes genes with metallopeptidase activity and one gene, GA10010, with a *D. melanogaster* orthologue, *drm*, that is a known zinc finger protein. It is possible that genetic conflict might have driven the faster evolution of the Ovd protein and that through its effects on the misregulation of a complex of genes reminiscent to the Spartan domain of GCNA, could contribute to alterations in chromatin condensation and packaging that result in HMS.

## Conclusions

Gene × gene interactions commonly underlie fitness disruptions in interspecies hybrids [6–12]. Several cases of disruption of gene expression in interspecies sterile hybrid have been documented, and the effects of *cis* and *trans* interactions quantified [21, 34, 63, 76–79]. While *cis*-regulatory changes are predominant contributors to gene expression divergence between species [45, 46, 80], we still lack on the identification of interactions between *cis*-targets and *trans*-regulatory proteins that might contribute to speciation. Identifying these interactions is important because they provide a molecular explanation for the classical Bateson–Dobzhansky–Muller model of negative allele interactions in hybrids [81]. Such gene × gene interactions can trigger a decline of fitness and restrict gene flow between diverging populations or species. Here we identify 52 putative targets of the HMS gene *Ovd* and unveil a *trans-cis* interaction network that contributes towards our understanding of the genetics of population differentiation and speciation.

## Methods

### Putative X-linked transcription factors and sequence divergence

We identified putative transcription factors within two previously identified HMS loci in the X chromosome of the *D. pseudoobscura* subspecies pair [4, 12]. Protein coding genes within a previously characterized HMS locus in the right arm of the X chromosome [4] were located using FlyBase (http://flybase.org). This HMS locus is found flanked between GA19954 and GA23845, with the coordinates XR_group6: 9462912..9510762. For the other locus, we inferred its genomic location from the markers (*yellow* and *X22*) used to flank the locus [12]. The *yellow* marker is an annotated gene whose genomic location is XL_group1e: 4239101..4244756 in the r3.04 *D. pseudoobscura* reference genome (http://flybase.org). For *X22*, we used the molecular marker primer sequences [12] to BLAST against the *D. pseudoobscura* reference genome and found its location to be XL_group1e:6561650..6561795. We then identified protein coding genes within the *X22-y* region of the left arm of the X chromosome (XL_group1e:4239101..6561795). We retrieved amino acid sequences for all protein coding genes within the two loci using FlyBase (http://flybase.org/) and searched for nucleic acid binding domains using default parameters within ScanProsite (https://prosite.expasy.org/scanprosite/).

For the identified putative transcription factors, sequence alignments of 31 *D. p. pseudoobscura* and 5 *D. p. bogotana* strains were downloaded from PseudoBase (http://pseudobase.biology.duke.edu/) [82] and used to reconstruct Neighbour Joining Poisson corrected protein trees using MEGA [83]. We looked for proteins that reliably clustered the two subspecies apart, with the reliability of the split assessed by bootstrapping with 1000 replicates [84]. For all proteins, we identified fixed amino acid substitutions or shared amino acid polymorphisms that defined subspecies-specific haplotypes.

We used two different methods to estimate the potential deleterious effect of fixed amino acid substitutions on protein function. PolyPhen-2 (http://genetics.bwh.harvard.edu/pph2/) uses a naïve Bayes classifier to derive information from sequence alignments and protein structural properties and predicts the effect of an amino acid substitution on the function of a protein. PolyPhen-2 scores near 1 are predicted to be more likely deleterious [85]. Provean (http://provean.jcvi.org) determines the impact of the amino acid substitution on protein function based on an alignment score. The effect on the protein query sequence (*D. p. pseudoobscura*) and its fixed variant change (*D. p. bogotana*) is tested with respect to sequence homologs collected from the NCBI nr protein database through BLAST [86]. To increase the sensitivity of detection of deleterious variants, we used a higher than default score threshold (*i.e*., −1.3).

### Fly stock maintenance

Stocks used in this study were obtained from the University of California San Diego (UCSD) *Drosophila* stock center: *D*. *p. pseudoobscura*, *sepia* (14011–0121.08) and *D. p. bogotana* (14011–0121.175). Stocks were maintained at 23 °C on a 12-h light/dark cycle. Fly colonies were cultured on cornmeal-yeast-agar medium. Virgin females were collected post-eclosion and mass matings were performed for introgressions and to generate F_1_ sterile hybrid males.

### Genetic introgression of *Ovd*

To identify genes possibly regulated by the state of the *D. p. bogotana* or *D. p. pseudoobscura* alleles of *Ovd,* we took advantage of the fact that the *Ovd* locus for *D. p. pseudoobscura* is tightly linked to the *sepia* eye gene [4]. This eye color mutation acts as a visible marker allowing the use of a backcross design to introgress the fertile *D. p. pseudoobscura* allele of *Ovd* (*Ovd*^*p*^) into an otherwise pure *D. p. bogotana* X chromosome. The introgression followed a previously described protocol [4]. Briefly, it started by crossing virgin *D. p. bogotana* females (14011–0121.175) with naïve *D. p. pseudoobscura* males with *sepia* eyes. Since recombination occurs in females and the marker is only visible in males, all F_1_ virgin females were collected and backcrossed with *D. p. bogotana* males to produce the next generation. F_2_ fertile males with *sepia* eyes were then selected and backcrossed with *D. p. bogotana* females. This alternating mating scheme, where female hybrids were collected in odd-numbered generations and male hybrids with the *sepia* eye color and the *D. p. pseudoobscura* allele of *Ovd* were collected in even-numbered generations, was continued for 27 generations. At the 27th generation, females were collected and backcrossed with the paternal *D. p. pseudoobscura sepia* strain to generate *sepia* and non-*sepia* eyed F_28_ hybrid males. Fertility was analyzed as a binary trait, with males considered sterile if they produced no offspring when paired with females from either subspecies. Due to the tight linkage between *Ovd* and *sepia,* hybrid males with *sepia* eyes have the *Ovd*^*p*^ fertile allele and are fertile. Non-*sepia* males have the *Ovd*^*b*^ X-linked allele, are sterile, and their genome is expected to be nearly identical to the genome of the F_1_ sterile male hybrids (Fig. 1).

### Testes RNA sample preparation and sequencing

Total RNA was extracted from 12–15 pairs of testes using the Aurum Total RNA Mini Kit (Bio-Rad). Three biological replicates were obtained for the parental subspecies, sterile F_1_ hybrid, and F_28_ *sepia* and non-*sepia* hybrid males (*i.e*., 15 samples). For each sample, RNA concentration and purity was determined using a NanoDrop spectrophotometer by examining the A260/280 and A260/230 ratios. Quality RNA samples were sent to the Génome Québec Innovation center (https://www.genomequebec.com/) for library preparation and sequencing. Before library construction, the quality of the samples was further verified using an Agilent Bioanlyser and the libraries were prepared using the NEBNext mRNA stranded library preparation kit. All 15 samples were ran multiplexed on a single lane of an Illumina HiSeq4000.

### Differential gene expression analysis

After sequencing, a quality check of the raw RNA-sequence data was performed using FastQC [87]. Read processing and adapter trimming were then performed with Trimmomatic [88] and reads with a Phred score below 30 and a final length less than 50 bp were excluded. Trimmed reads were then mapped to r3.04 of the *D. p. pseudoobscura* reference genome (http://flybase.org/) using STAR [89] under default settings. After mapping, read counting was performed using featureCounts [90] at the gene level with the reversely stranded (−s 2) and fragment counting (−p) parameters and r3.04 of the *D. p. pseudoobscura* annotation serving as a guide.

Pairwise differential expression analyses across the parental subspecies, their F_1_ sterile male hybrid, and the F_28_ fertile (*sepia*) and sterile (non-*sepia*) hybrid males from the introgression were performed using DESeq2 [91] and edgeR [92] which both use the negative binomial distribution model in their analyses. For the edgeR analysis, a minimum count-per-million (CPM) value of 1, which is equivalent to at least 10 counts, was used for filtering to avoid bias toward genes expressed in larger libraries [93]. Per gene counts for each sample were normalised using the TMM method [94]. In the analysis using DESeq2, the local fit type was used and the independent filtering method was performed. We used an FDR of 5% and a lfc threshold higher than 1 was further applied to both edgeR and DESeq2 results to increase the true positive rate [95] and the consensus list of differentially expressed genes between these tools was used for all downstream analyses. All tools used for the differential gene expression analysis were ran on UseGalaxy (https://usegalaxy.org/). Potential targets of *Ovd* are genes differentially expressed between F_28_ *sepia* and non-*sepia* hybrid males, while targets of *Ovd* more likely associated with hybrid male sterility are common genes differentially expressed in both F_28_ non-*sepia* hybrid male and F_1_ sterile hybrid male samples relative to both parental subspecies.

### Chromosomal distribution, non-coding sequence, and allele-specific expression divergence for targets of Ovd

We used the PseudoBase JBrowse tool (http://pseudobase.biology.duke.edu/) [82] to retrieve the chromosomal locations of the identified targets of Ovd and considered genes as members of a cluster if they were within 5Kb of each other. We used PseudoBase to retrieve *D. p. pseudoobscura* and *D. p. bogotana* sequences 3000 nucleotides upstream and 200 downstream (−3000 to +200) of the transcription start site (TSS) of targets of Ovd. The sequences were aligned using MUSCLE within MEGA [83]. Both polymorphisms within, and fixed substitutions between subspecies were identified using DnaSP [96]. Within the alignments, we searched for Adf-1 transcription factor binding sites, a putative target for the MADF domain found in the Ovd protein, using PROMO (PROMO: http://alggen.lsi.upc.es/cgi-bin/promo_v3/promo/promoinit.cgi?dirDB=TF_8.3/) [97, 98]. Polymorphisms and fixed changes within Adf-1 sites were identified using MEGA.

We determined the relative contribution of *cis-* and *trans-*regulatory divergence on gene expression of targets of Ovd by identifying fixed subspecies-specific SNPs and relative allelic expression in the hybrid [34, 77]. SNPs between the parental subspecies were identified from their mapped reads using Naïve variant caller followed by processing with Variant annotator [99]. SNPs were considered fixed in each parental subspecies if each parent had a single different allele and at least 3 supporting reads. We assigned hybrid RNA-seq reads to a parent of origin based on the identity of the allele at fixed SNP positions in each parent. Reads with fixed SNPs mapping to a gene were counted and any gene with at least 20 reads mapped to parental subspecies were retained [34, 77]. SNP counts for each gene were adjusted to account for differences in sequencing depth between samples and samples with zero SNP counts were given a value of 1 to allow for statistical testing. Relative contributions of mapped reads were calculated and significant differences in expression between parents (P_pse_ vs P_bog_, using a binomial exact test), between parental alleles in the sterile (non-*sepia*) F_28_ hybrid (H_pse_ vs H_bog_, using a binomial exact test) and between the ratio of parental read counts to counts of each parental allele in sterile (non-*sepia*) F_28_ the hybrid (P_pse_/P_bog_ vs H_pse_ vs H_bog_, using Fisher’s exact test), were determined. FDR corrected q-values were used for all three tests (significance q < 0.5%). We identified patterns of regulatory evolution for each target of Ovd according to the outcome across the three statistical tests implemented [34, 77], namely:

- Conserved: No detectable divergence between *cis-* and *trans-*regulatory elements. No significant difference in expression between parental subspecies (P_pse_ = P_bog_) and between parental alleles in the hybrid (H_pse =_ H_bog_). No significant difference between the ratio of parental allele expression and the ratio of parental alleles in the hybrid (P_pse_/P_bog_ = H_pse_/H_bog_).
- *Cis*-only: Divergence in a *cis-*regulatory element. Significant difference in expression between parental subspecies (P_pse_ ≠ P_bog_) and between parental alleles within the hybrid (H_pse_ ≠ H_bog_). No significant difference between the ratio of parental expression and the ratio of parental alleles in the hybrid (P_pse_/P_bog_ = H_pse_/H_bog_).
- *Trans-*only: Divergence in a *trans*-regulatory element. Significant difference in expression between the parental subspecies (P_pse_ ≠ P_bog_) but not between the parental alleles within the hybrid (H_pse_ = H_bog_). Significant difference between the ratio of parental expression and the ratio of parental alleles in the hybrid (P_pse_/P_bog_ ≠ H_pse_/H_bog_).
- *Cis* and *trans*: Regulatory divergence is detected between both *cis-* and *trans-*regulatory elements. Significant differences are observed between parental subspecies expression (P_pse_ ≠ P_bog_), between parental alleles within the hybrid (H_pse_ ≠ H_bog_), and between the ratio of parental expression and the ratio of parental alleles in the hybrid (P_pse_/P_bog_ ≠ H_pse_/H_bog_).
- Compensatory: Regulatory divergence is detected in both *cis-* and *trans-*regulatory elements but they perfectly compensate each other. This results in no observable difference between parental subspecies expression (P_pse_ = P_bog_). Significant difference in expression between parental alleles in the hybrid (H_pse_ ≠ H_bog_) and between the ratio of parental expression and the ratio of parental alleles in the hybrid (P_pse_/P_bog_ ≠ H_pse_/H_bog_).
- Ambiguous: Patterns of statistical results that do not fall into any of the above categories.
- We then broadly classified *cis*-only, *cis* and *trans*, and compensatory into a group of genes showing *cis* effects.

## Supplementary Information

**Additional file 1: Figure S1.** Subspecies-specific haplotypes of GA22224 resolve *D. p. pseudoobscura* and *D. p. bogotana* apart. A: Neighbour Joining Poisson corrected protein tree showing *D. p. bogotana* (boxed) strains clustered separately from *D. p. pseudoobscura* strains. Bootstrap values are shown. B: Protein alignment showing variable amino acid sites. *D. p. bogotana* (D.p. bog) strains names are bolded and the subspecies-specific haplotypes are shown in grey.

**Additional file 2: Table S1.** Number of raw, trimmed, and uniquely mapped reads along with the mapping rate for each sample. **Table S2.** Expression of Ovd gene targets (in normalised counts and CPM for DESeq2 and edgeR respectively) and the FDR-corrected p-values for each pairwise comparison relative to parental species. Classification of misregulation is determined by the consensus result of both differential expression tools used. Genes misregulated in both sterile conditions and with additive or non-differential expression between the F28 fertile and parentals are highlighted as putative sterility-targets. **Table S3.** Chromosome distribution of Ovd targets with sterility targets Gene IDs bolded. The gene symbol of gene clusters are highlighted in yellow along with the distance between genes in the cluster. The location of genes within previously mapped autosomal QTLs is highlighted in grey. **Table S4.** BLASTp results showing putative D. melanogaster orthologs of genes within clusters. *D. melanogaster* genes with probability of random match lower than 0.01 are bolded. **Table S5**. Potential *cis* changes among mapped targets of Ovd. ‘Adf-1 sites’ is the total number of putatitive binding sites found 3000 bp upstream of the TSS. ‘Total_pol’ is the total number of polymorphism found at Adf-1 sites. ‘Fixed’ refers to fixed nucleotide changes between *D. p. pseudoobscura* and *D. p. bogotana* at identified Adf-1 sites. For one gene the combination of polymorphisms allows differentiation between the subspecies. ‘ASE’ is the result of allele specific expression and ‘Mode’ differentiate genes that show some form of *cis*-regulation form others. Fixed substitutions is the total number of fixed nucleotide changes between subspecies in the −500 to +200 (500), −1000 to +200 (1000), −2000 to +200 (2000) and − 3000 to +200 (3000) gene regions.

## Data Availability

All data generated and analyzed during this study are included in the supplementary information files. All databases used in this study are open. FlyBase (https://flybase.org/) was used for retrieval of amino acid sequences of proteins located within X-linked HMS loci and ScanProsite (https://prosite.expasy.org/scanprosite/) to search for presence of nucleic acid binding domains. PseudoBase (http://pseudobase.biology.duke.edu/) was used to retrieve gene targets of Ovd chromosomal location and their sequence alignments of different strains of *D. p. pseudoobscura* and D*. p. bogotana*. Polyphen-2 (http://genetics.bwh.harvard.edu/pph2/) and Provean (http://provean.jcvi.org) were used to test deleterious effects of amino acid substitutions on protein function. The BLAST tools within GenBank (https://blast.ncbi.nlm.nih.gov/Blast.cgi) were used to identify HMS loci genome boundaries and to search for *D. melanogaster* orthologs of targets of Ovd. PROMO (http://alggen.lsi.upc.es/cgi-bin/promo_v3/promo/promoinit.cgi?dirDB=TF_8.3/) was used to identify Adf-1 transcription binding sites among targets of Ovd. g:GOST within g:Profiler (https://biit.cs.ut.ee/gprofiler/gost) was used for functional enrichment analysis among gene targets of Ovd. All raw transcriptome data reported in this article have been deposited in the Sequence Read Archive (SRA) (https://www.ncbi.nlm.nih.gov/sra) under accession numbers SRR14693691, SRR14693700, SRR14693699, SRR14693698, SRR14693697, SRR14693696, SRR14693695, SRR14693694, SRR14693693, SRR14693692, SRR14693705, SRR14693704, SRR14693703, SRR14693702, and SRR14693701. All tools used for gene expression analysis were run on UseGalaxy (https://usegalaxy.org/).

## Abbreviations

ASE: Allele specific expression
BLAST: Basic local alignment search tool
CPM: Counts per million
FDR: False discovery rate
HMS: Hybrid male sterility
lfc: log_2_ fold change
MEGA: Molecular evolutionary genetics analysis
MUSCLE: MUltiple sequence comparison by log-expectation
*Ovd*: *Overdrive*
*Ovd*^*b*^: *D. p. bogotana* allele of *Overdrive*
*Ovd*^*p*^: *D. p. pseudoobscura* allele of *Overdrive*
QTL: Quantitative trait locus
SNP: Single nucleotide polymorphism
TMM: Trimmed mean of M-values
TSS: Transcription start site
